# Cushny’s Pharmacology and Therapeutics

**Published:** 1903-06

**Authors:** 


Cushny's Pharmacology and Therapeutics. A Textbook of Pharmacology and Therapeutics; or the Action of Drugs in Health and Disease. By Arthur R. Cushny, A. M., M. D., Professor of Materia Medica and Therapeutics, University of Michigan, Department of Medicine and Surgery, Ann Arbor, Mich. Third edition, revised and enlarged. In one 8vo volume of 750 pages, with 52 engravings. Philadelphia and New York: Lea Brothers & Co. 1903. [Cloth, $3.75 net; leather, $4.75 net.]

If one looks for the causes of current scepticism as to the value of drugs and of carelessness in their application, a great deal must be charged to ignorance of the precise action of the more important agents. Our current therapeutic literature is too dogmatic and our practice too empirical. But our teaching of therapeutics is improving, thanks to such books as the one before us. American medicine today owes a debt of gratitude to Schmiedeberg and his pupils in this country, who are working for the development of sound therapeutics upon a basis of exact pharmacology. The dedication of this book to Oswald Schmiedeberg is therefore appropriate. While Cushny's pharmacology presents a radical departure from the style of most current therapeutic literature, in that laboratory experiment is made the basis of study and application of drugs, the appearance of a third edition within four years is evidence that just such a departure is welcomed.

The chief criticism that is likely to be offered is that the work is not sufficiently practical for the physician's use. The validity of this objection depends upon the viewpoint of the critic. In the reviewer's opinion the most important needs of physicians and students today in the field of therapeutics, is a more thorough knowledge of drugs and their definite effects and the ability to apply the remedy to the known pathological condition. To meet these needs this book is well adapted, and from the teacher's point of view is to be commended, both as to scope and detail. It is very readable, being unsurpassed in diction and clearness of statement by any book of its class in the English language. It gives what is actually known, and the best of what is believed, concerning the action and value of drugs. The discussions of doubtful points are truly scientific and happily exclude verbose rehearsals of historic experiments.

The illustrations are useful in the highest degree, as they usually represent actual experiments. Of special importance are the illustrations of the action of caffeine, digitalis, anesthetics and alcohol. The present edition gives much additional matter concerning alcohol and brings to us conclusions that are likely to stand as our estimate of that much discussed drug, for some time to come. To the advanced student in therapeutics is the book to be especially commened. Good binding and attractive form, with its superior character contributed by the author, make it a book that ranks with the very best in its line.

